# Dietary Patterns May Be Nonproportional Hazards for the Incidence of Type 2 Diabetes: Evidence from Korean Adult Females

**DOI:** 10.3390/nu11102522

**Published:** 2019-10-18

**Authors:** Sangwon Chung, Myung Sunny Kim, Chang Keun Kwock

**Affiliations:** 1 Korea Food Research Institute, 245, Nongsaengmyeong-ro, Iseo-myeon, Wanju-gun, Jeollabuk-do 55365, Korea; schung@kfri.re.kr (S.C.); truka@kfri.re.kr (M.S.K.); 2 Department of Food Biotechnology, Korea University of Science & Technology, Wanju 55365, Korea

**Keywords:** diabetes, dietary patterns, proportional hazards, cox regression, metabolic syndrome

## Abstract

This study aimed to examine the association between the incidence of type 2 diabetes and various risk factors including dietary patterns based on the rigorous proportional hazards assumption tests. Data for 3335 female subjects aged 40–69 years from the Korea Genome and Epidemiology Study were used. The assumption of proportional hazards was tested using the scaled Schoenfeld test. The stratified Cox regression was used to adjust the nonproportionality of diabetic risk factors, and the regression was adjusted for potential confounding variables, such as age, marital status, physical activity, drinking, smoking, BMI, etc. Metabolic syndrome and meat and fish pattern variables were positively associated with diabetes. However, dietary patterns and metabolic syndrome variables violated the proportional hazards assumption; therefore, the stratified Cox regression with the interaction terms was applied to adjust the nonproportionality and to allow the possible different parameters over each stratum. The highest quartile of meat and fish pattern was associated with diabetes only in subjects aged over 60 years. Moreover, subjects who were obese and had metabolic syndrome had higher risk in bread and snacks (HR: 1.85; 95% CI: 1.00–3.40) and meat and fish pattern (HR: 1.82; 95% CI: 1.01–3.26), respectively. In conclusion, a quantitative proportional hazards assumption test should always be conducted before the use of Cox regression because nonproportionality of risk factors could induce limited effect on diabetes incidence.

## 1. Introduction

Diabetes is a chronic disease that has serious health consequences such as heart attack, stroke, loss of sight, and renal failure. The prevalence of diabetes is growing faster than ever worldwide and is growing faster in low- and middle-income countries than in high income countries [1]. Zhang et al. [2] estimated that 12% of global health expenditures would have been spent on diabetes in 2010 and these expenditures are expected to grow to USD 490 billion by 2030.

Due to the medical and economic significance, the associations between diabetes and various risk factors have been widely examined; the risk factors include diet, obesity, metabolic syndrome (MS), drinking, family history, etc. Among these risk factors, a large number of studies have assessed the relationship between diet and type 2 diabetes (T2D) using dietary patterns (DPs), because DPs reflect characteristics of the overall diet of individuals [3]. In a meta-analysis of fifteen cohort studies, higher adherence to healthy DP, such as high intake of whole grains, fruits, vegetables, and low-fat dairy, was significantly associated with a 21% reduced risk of T2D when compared to lower adherence to healthy DP [3].

The primary workhorse of many cohort studies has been the Cox proportional hazards (PH) regression model. Even though this regression model is readily available in statistical packages, such as SAS, STATA, and R., it specifically requires that the underlying hazard functions for any levels of independent variable be proportional over the entire observation period [4]. If this assumption is not met, then the parameters being estimated by the Cox regression model may not be meaningful at all [5]. According to authors’ analysis on the frequency of implementation of the proportionality test, using the references from a meta-analysis done by Jannasch et al. [6], which reviewed only prospective studies to examine the association between DP and T2D, few studies implemented the proportionality test of the hazard. Among the 11 papers extracted for a meta-analysis, only two papers done by de Koning et al. [7] and Otto et al. [8] actually implemented the proportionality test. Even though the assumption of proportionality is a prerequisite for using Cox proportional regression, the test of proportionality is not carried out in many studies before the use of the regression.

Thus, the aim of this study is to examine the association between incident T2D and various risk factors including DP, after rigorous proportionality tests using Korean prospective cohort data. The reason we pay attention to the Korean diabetes issue is that Asians are more susceptible to diabetes at a given body mass index (BMI) [9], and Asian studies are needed due to the ethnic disparity despite plentiful study in this area [10,11]. Particularly in Korea, the diabetes problem has deteriorated. According to Korean Diabetes Association [12], prevalence of diabetes was 12.4% in 2011 and increased to 14.4% in 2016 among aged 30 or over. The prevalence of men was 14.5% and that of women was 10.4% in 2011, and the prevalence of men was 15.8% and that of women was 13.0% in 2016. The difference of prevalence between men and women was 4.1% point in 2011 and 2.8% in 2016, indicating that the prevalence of women rapidly increased [12]. In addition, it has been observed that unhealthy dietary patterns, such as ‘Bread and Meat and Alcohol’ pattern was associated with high cholesterol levels among Korean adults with diabetes [13]. We also look for potential interaction effects among diabetic risk factors because omitting significant interaction terms causes bias in parameter estimation statistically, and a significant interaction between predictors could lead to particular public health implications from an epidemiologic perspective [14].

## 2. Materials and Methods

### 2.1. Study Population

We used data of subjects from Korea Genome and Epidemiology Study (KoGES). KoGES is a consortium of six prospective cohort studies to investigate dietary, lifestyle, genetic, and environmental determinants associated with non-communicable chronic diseases such as obesity, cardiovascular diseases, hypertension, hyperlipidemia, and T2D and their association among Koreans. This study used data of a community-based cohort study, the Ansan and Ansung study among the six cohorts. Ansan and Ansung are average urban and rural regions in Korea, respectively. Detailed information of the study is described in a previous study [15]. The KoGES Ansan and Ansung study, a large population-based study, recruited Korean adults who were aged 40 to 69 at baseline from 2001 to 2002. The survey consists of socio-demographic, lifestyle, and health questionnaires, dietary interview, and anthropometric and clinical measurements and a follow-up survey has been conducted biennially up to the seventh follow-up. This study was exempt from review by the Institutional Review Board at the Korean National Institute for Bioethics Policy (IRB exemption number: P01-201702-21-004).

Among 10030 subjects at baseline, those who had T2D or did not have information on T2D at baseline (*n* = 1253); who did not participate in the follow-up surveys (*n* = 843); who were diagnosed with or having treatment or medication for hypertension, hyperlipidemia, and cardiovascular diseases (*n* = 1316); who did not have dietary data (*n* = 206); and who had implausible energy intake (<500 or >5000 kcal/day, *n* = 60) were excluded. Among these subjects, we only included data for 3335 female subjects for the analysis based on the evidence that women with diabetes are more susceptible to the incidence of other chronic diseases, such as heart failure, than men in Korean population [16].

### 2.2. Dietary Assessment

Dietary intake data were collected by validated semiquantitative food frequency questionnaire (FFQ) [17] at baseline and the second follow-up interview by trained staff. The FFQ consisted of 103 food items and was classified into nine categories of frequency: never or rarely, once a month, 2–3 times a month, 1–2 times a week, 3–4 times a week, 5–6 times a week, once a day, 2 times a day, and 3 times a day. The options for portion size were 1/2 serving, 1 serving (standard), and ≥2 servings. The amount of food intake a day was calculated by multiplying the food intake frequency and the food intake at a time. The FFQ survey was conducted only twice, at baseline and second follow-up. We used the data at baseline for subjects who acquired T2D or who were censored up to the first follow-up, and the data at the second follow-up for subjects who acquired T2D or who were censored after the second follow-up.

### 2.3. Outcome Ascertainment

The primary outcome was a diagnosis of T2D. The subjects were diagnosed with diabetes if any one of the following criteria based on the health questionnaire answers and blood test results were satisfied: (1) self-reported diabetes diagnosed by a physician; (2) receiving medication for diabetes or insulin for more than three months; and (3) fasting glucose level ≥126 mg/mL or 2 h after glucose load ≥200 mg/dL (oral glucose tolerance test result).

### 2.4. Othe Covariates

Socio-demographic, life-style, and health-related factors were used as covariates. For socio-demographic factors, age, marital status, household income, and education were included. Age was divided into 3 categories: 40–49 years, 50–59 years, and ≥60 years at baseline. Marital status was divided into 2 categories: lives with a spouse and single including unmarried, separated, divorced, and widowed status. Household income was divided into 2 categories: high income (≥840 dollar/month) and low income (<840 dollar/month). Education was divided into 2 categories: below high school graduate and high school graduate or above. For life-style and health-related factors, physical activity, drinking, family history of T2D, MS, and BMI were included. Physical activity was obtained from the answer to the question “How long do you do vigorous exercise a day?” and categorized into 2 groups: <30 minutes/day and ≥30 minutes/day. Likewise, MS was categorized into 2 groups, ‘≥3 factors satisfied’ (yes) and ‘<3 factors satisfied’ (no), and diagnosed with the following definitions of five components: (1) waist circumference ≥85 cm for women; (2) SBP ≥130 mmHg or DBP ≥85 mmHg; (3) TG levels ≥150 mg/dL; (4) HDL cholesterol levels <50 mg/dL for women; and (5) fasting plasma glucose levels ≥100 mg/dL. The definition was based on the National Cholesterol Education Program Adult Treatment Panel Ⅲ [18] and the standard of waist circumference was based on the standard reference for Koreans [19].

### 2.5. Statistical Methods

All statistical analyses were conducted across the quartiles of each DP score using STATA S/E 15.0 (StataCorp, College Station, TX, USA). The comparisons of general characteristics of subjects at baseline were assessed by the chi-square test for categorical variables and one-way ANOVA for continuous variables. Multivariate adjusted Cox PH regression models were used to assess hazard ratios (HRs) and 95% confidence intervals (95% CIs) of incident T2D. All health-related, life-style, socio-demographic, and dietary variables including DP score and calorie intake were included in the modelsDPs were derived from principal-component factor analysis by using the intake of food weight by 32 predetermined food groups. We categorized 103 food items into 32 food groups based on nutritional characteristics and similarities in ingredients for factor analysis. The 32 food groups we categorized are shown in Table 1. To achieve a simpler structure and to enhance the interpretability of factors, an orthogonal varimax rotation was implemented. We identified three factors to be retained for DPs of subjects based on a Scree plot, the eigenvalue (>1.75), and interpretability of factors. The name of the dietary pattern was determined by the food groups having an absolute factor loading more than 0.3.

Cox PH regression models assume that the HR is constant over time; therefore, it is very important to evaluate the validity of the assumption. The rigorous statistical method of evaluating the proportionality assumption is to test nonzero slope in a generalized linear regression of the scaled Schoenfeld residuals on time [20]. The scaled Schoenfeld residuals test was used to test the null hypothesis of zero slope, and the rejection of the null hypothesis indicates the violation of the PH assumption [21]. The scaled Schoenfeld residuals tests identified that MS status and DP variables violated the proportionality assumption of Cox regression.

To handle the nonproportionality problem for the MS and DP variables, stratified Cox regression was used [22]. The Cox regression stratified by the categories of MS (2 categories) and DP variables (4 categories), total of 8 strata, was estimated. This stratified Cox regression assumes the same parameter estimates for the 8 strata; therefore, this should be tested. To test the equal parameters over all 8 strata, we allowed interaction terms between covariates with PH and categories of covariates with nonproportional hazard (NPH) excluding the reference category in the Cox regression. The maximum number of the interaction terms is 77 terms; the number of binary variables identifying 8 strata, 7; times the number of proportional covariates, 11. Among these 77 interaction terms, we excluded the 33 interaction terms multiplying three discrete variables; for example, MS × Q2 (the second quartile of each dietary pattern) × age ≥60 years, because they have very similar values and cause the multicollinearity problems in estimation.

## 3. Results

### 3.1. Classification of Dietary Patterns

Three DPs were identified: bread and snacks, meat and fish, and Korean traditional patterns. The detailed description of food groups and the factor loading scores of the DPs in this study are shown in Table 1. These three DPs explained 29.8% of the total variation in food intake.

### 3.2. Characteristics of Study Subjects

General characteristics of the study subjects at baseline are shown in Table 2. The subjects in the highest quartile of the bread and snacks pattern group were more likely to be younger, to live with a spouse, to have higher income and higher education levels, and to drink whereas these individuals were less likely to be physically active. In the meat and fish pattern, the highest quartile group was more likely to have higher income and higher education levels. In addition, this group was less likely to be physically active, to have family members who were diagnosed with T2D. Finally, the highest quartile group of the Korean traditional pattern was likely to be younger, to have lower income and lower education levels, and less likely to be physically active, and to drink. Among health-related factors, the highest quartile group of the Korean traditional pattern was more likely to be obese and to have MS. Energy and major nutrient intakes were significantly different by the quartiles of each DP.

### 3.3. Proportionality of Risk Factors

Three Cox regression models for each DP were estimated with identical diabetic risk factors under the assumption of PH. Table 3 shows the Cox regression estimates for the three DPs under the assumption of PH. Compared to the reference groups in the all DPs, subjects who were in their 50s and who had family history of T2D, MS, or obesity showed significantly increased risk of T2D. In addition, the risk of T2D in the highest quartile of meat and fish pattern was 72% higher than that in the lowest quartile group (HR: 1.72; 95% CI: 1.28–2.32).

Our results look similar to those of previous studies in that age, family history of T2D, MS, and obesity are the significant risk factors of incidental diabetes. In addition, the DP variables show mixed results in that bread and snacks and Korean traditional patterns do not have a significant effect on the incidence of diabetes, while the meat and fish pattern has a significant positive effect. However, since the Cox regression was estimated under the assumption of proportionality, the parameters estimated can be meaningless if the assumption is not met. Therefore, a rigorous test of proportionality, the scaled Schoenfeld test, is required.

Table 4 shows the scaled Schoenfeld test result for each predictor variable. Significant *p*-values represent the rejection of the null hypothesis of PH for the specific predictor variables. According to the scaled Schoenfeld tests of proportionality, MS, the highest quartile group of the bread and snacks DP, and the third and highest quartile groups of meat and fish and Korean traditional DP variables were suspicious of NPH. Therefore, the stratified Cox regression was employed to adjust the two predictors, MS and DP, which did not satisfy the proportionality assumption.

### 3.4. Stratified Cox Regression with Interactions between Risk Factors

We reported the stratified Cox regression result for meat and fish pattern only in Table 5, since results for other dietary patterns were very similar and we were able to present the significance of our result.

The parameter estimates from the stratified Cox model with interaction terms allowing different parameter estimates are shown in Table 5. We further investigated to look for the significant interaction terms because the parameters estimated from the misspecified models without interaction terms are biased [14]. We found the significant interaction effects between age variable (Age ≥ 60 years) and the highest quartile of meat and fish dietary pattern (Q4) (HR: 2.86; 95% CI: 1.12–7.33) and between obesity (BMI ≥ 25) and MS in stratified Cox model with meat and fish (HR: 1.82; 95% CI: 1.01–3.26) patterns. From these results, we were able to obtain different epidemiological implications from the general Cox regression results: (1) high meat and fish pattern score increases the risk of T2D to the subjects in their 60s; (2) MS variable raises risk of T2D only to obese subjects.

## 4. Discussion

This study has examined the proportionality of risk factors for the incidence of T2D with the rigorous quantitative test of PH assumption and observed that nonproportionality of risk factors for T2D was identified using the data of Korean women aged 40–69 who participated in the KoGES study. The proportionality assumptions for MS and DP variables were violated for the incidence of T2D while age, marital status, household income, education, physical activity, drinking, family history of T2D, BMI, and energy intake are proportional to the incidence of T2D. To adjust for the nonproportionality of MS and DP, we stratified our analysis on these variables and allowed some interaction terms. As a result, positive and interactive associations of DP score and age over 60 years, and MS and obesity with the incidence of T2D were found. The subjects in their 60s and the highest quartile of meat and fish pattern had almost threefold higher risk of T2D, and obese subjects with MS had a higher risk in the stratified Cox model with meat and fish patterns. DPs are associated with diabetes; however, our findings suggest that DP is a risk factor for the incidence of diabetes only through interactive effects with age.

A meta-analysis has revealed that unhealthy diets comprising processed meat, refined grains, and fried products were associated with a higher risk of diabetes [6]. In addition, a meat DP including high consumption of red meat, red meat byproducts, high-fat red meat, and other seafood increased the risk of MS in a Korean study [23]. In a Singapore and Chinese adult study, a dim sum and meat-rich pattern increased the risk of T2D in never-smoker groups [24]. Our finding that the meat and fish pattern is associated with the incidence of T2D is consistent with the above studies. Moreover, a Korean traditional pattern, deemed to be a healthy diet in obesity, did not have any interactive effect on the incidence of T2D. This finding also confirmed that a healthy DP did not have protective effect on type 2 diabetes [25,26,27]. Lorenzo, et al. [28] had found that obesity worsens the risk of diabetes with MS, and this finding is also equivalent to our finding that interaction effect between obesity and MS raises the risk of incidence of T2D.

The striking difference of the present study is that meat and fish pattern is a nonproportional hazard and this led to the different epidemiological implication: high meat and fish pattern score is a significant hazard only to the subjects aged 60 or over. We can conjecture the reason behind this finding: unhealthy dietary patterns are linked not only to obesity but to the unhealthy life style, such as smoking, drinking, sedentary lifestyle, etc., and these risk factors increase the accumulation of inflammation and oxidative stress in the body over lifetime span [29,30]. Especially, evidence regarding relationship between meat consumption and inflammation and oxidative stress is accumulating [31,32]. Of course, it was documented that inflammation and oxidative stress in the adipose tissue increase angiotensinogen production and this led to elevated blood pressure and reduced insulin metabolic signaling [33].

This study has a few limitations. First, we only examined the association among women in this study due to the rapid increase in prevalence of diabetes in women. Further study is required to assess the association between diabetes incidence and its risk factors with rigorous proportionality tests among both sexes. Second, using FFQ could result in low accuracy and potential recall bias due to a closed-ended questionnaire and limited options for frequency and portion size, even though FFQ is the most suitable dietary assessment method for prospective studies [34]. However, the FFQ used in KoGES has already been validated for the survey [17], and the association between diet and diabetes has been widely examined through several studies from dietary data of KoGES [35,36]. Third, even though this study followed up the incidence of diabetes for 14 years, dietary intake data used in this study were only obtained at the baseline and the second follow-up. This might not have exactly linked DPs and the incidence of diabetes. Despite these limitations, we examined the PH assumption for diabetes risk factors, identified the NPH issues among risk factors, and made adjustments for the violation of PH assumption. To our knowledge, this is the first study which tests the proportionality assumption of covariates and actually reports the nonproportionality of DP risk in a large, population-based Korean cohort study. 

In conclusion, when the PH assumption is violated, the usual HR is not correct [37]; therefore, adjustment should be made. As we allowed interaction terms in a multivariable Cox regression model, we found not only significance of interaction terms but also specific interaction effects between PH and NPH variables. The existences of some significant interaction terms between PH and NPH variables mean that the different HRs for PH variables should be reported for each NPH stratum. Since there is no guarantee that this assumption will hold, especially in a large prospective study, this should be checked and fixed if the issues are present. Moreover, further cohort studies are warranted to confirm our results.

## Figures and Tables

**Table 1:** Food groups and items from semiquantitative FFQ for dietary patterns and rotated factor loadings ^a^ of the identified three dietary patterns.

Food Groups	Food Items	Three Identified Dietary Patterns		
		Bread and Snacks	Meat and Fish	Korean Traditional
Cooked rice	Cooked white rice, and cooked rice with soybeans or various grains			0.50
Other grains	Rice cakes, cereals, corn flakes, and cereal powder	0.49		
Ramen	Ramen			
Chinese noodles	Chinese-style noodles with black bean sauce, Chinese-style noodles with vegetables and seafood	0.30		
Other noodles	Wheat noodles with soup and buckwheat noodle	0.40		
Dumplings	Dumplings, dumpling soup, and rice-cake soup	0.40		
Starch, potatoes, and sweet potatoes	Starch jelly, potatoes, and sweet potatoes			0.38
Bread, pizza, and hamburgers	Loaf bread, toast, bread with small red beans, sandwich, pizza, hamburgers, and other breads	0.62		
Snacks and sweets	Cakes, cookies, crackers, snacks, candy, chocolate, jam, honey, butter, and margarine	0.47		
Eggs	Eggs			0.42
Nuts	Peanuts, almonds, and pine nuts			
Legumes	Beans, beans cooked with soy sauce, and tofu			0.49
Soybean paste	Soybean paste and soup with soybean paste			0.57
Kimchi	Cabbage kimchi, radish kimchi, radish kimchi with water, and green onion kimchi			0.69
Yellow-green vegetables	Spinach, green pepper, zucchini, cucumber, broccoli, cabbage, lettuce, leeks, pepper leaves, perilla leaves, carrot, and carrot juice		0.50	0.48
Other vegetables	Radish, onions, white root (e.g., deoduck, doraji), bean sprouts, bracken, sweet potato stalk, and stem of taro		0.32	0.54
Mushrooms	Several types of mushrooms	0.37		0.37
Pickles	Korean vegetable pickles (garlic, garlic flower stalk, and radish)			0.45
Red meat	Pork belly, roasted pork, braised pork, steak, roasted beef, beef soup, dog meat, and red meat byproduct		0.81	
White meat	Fried chicken and chicken soup		0.68	
Processed meat	Ham and sausages		0.61	
Lean fish	Raw fish, hair tail, eel, yellow croaker, sea bream, flat fish, Alaskan pollack, and dried anchovies		0.72	
Fatty fish	Mackerel, Pacific saury, and Spanish mackerel		0.57	
Processed fish	Canned tuna, fish paste, and crab-flavored paste	0.53		
Salt-fermented fish	Salt-fermented fish	0.35		
Other seafood	Squid, octopus, clams, whelk, oyster, crab, and shrimp	0.52	0.33	
Seaweed	Laver, kelp, and sea mustard	0.33	0.35	
Milk and yogurt	Milk and yogurt	0.34		
Other dairy products	Cheese and ice cream	0.42		
Fruits	Strawberries, melon, muskmelon, watermelon, peaches, plums, bananas, persimmons, tangerines, pears, apples, oranges, grapes, tomatoes, cherry tomatoes, and several types of fruit juices	0.51		
Tea	Green tea	0.30		
Coffee and other drinks	Soybean, coffee, coffee with sugar, coffee with cream, carbonated drinks, and other drinks	0.34		
Variance of intake explained (%)		10.4	10.3	9.1

^a^ Factor loadings are shown if an absolute factor loading is more than 0.3.

**Table 2:** Baseline characteristics of female subjects according to dietary patterns.

Variables	Bread and Snacks				*p*-Value ^a^	Meat and Fish				*p*-Value ^a^	Korean Traditional				*p*-Value ^a^
	Q1	Q2	Q3	Q4		Q1	Q2	Q3	Q4		Q1	Q2	Q3	Q4	
Age (%)					<0.001					0.197					<0.001
40–49 years	29.0	50.3	67.0	69.9		53.3	51.4	56.1	53.0		61.0	53.1	52.4	45.9	
50–59 years	30.4	25.4	21.4	21.0		25.0	23.8	23.4	26.5		20.8	24.2	26.0	28.4	
≥60 years	40.6	24.3	11.7	9.1		21.8	24.8	20.5	20.6		18.2	22.7	21.6	25.7	
Marital status (%)					<0.001					0.066					0.087
Lives with a spouse	82.1	86.3	90.2	91.6		86.2	86.9	86.3	89.9		86.2	85.9	89.3	88.7	
Single	17.9	13.7	9.8	8.4		13.8	13.1	13.7	10.1		13.8	14.1	10.7	11.3	
Household income ^b^ (%)					<0.001					0.010					<0.001
High income	38.0	59.1	76.9	80.5		60.2	62.7	67.7	61.3		68.9	62.2	64.6	55.5	
Low income	62.0	40.9	23.1	19.5		39.8	37.3	32.3	38.7		31.1	37.8	35.4	44.5	
Education (%)					<0.001					0.002					<0.001
Below high school graduate	88.4	71.2	52.4	43.5		68.2	64.5	59.4	66.3		54.6	68.1	65.1	71.2	
Above high school graduate	11.6	28.8	47.6	56.5		31.9	35.6	40.6	33.7		45.4	31.9	34.9	28.8	
Physical activity ^c^ (%)					<0.001					<0.001					<0.001
<30 min/day	45.5	70.8	77.2	75.6		62.5	70.2	76.4	58.4		73.9	71.1	65.3	55.3	
≥30 min/day	54.5	29.2	22.8	24.4		37.6	29.8	23.6	41.7		26.1	28.9	34.8	44.7	
Drinking (%)					<0.001					0.336					0.029
Nondrinker	78.4	72.8	68.6	67.3		69.5	72.5	71.8	73.5		68.3	74.4	72.9	72.3	
Drinker	21.6	27.2	31.4	32.7		30.5	27.5	28.3	26.5		31.7	25.6	27.1	27.7	
Family history of type 2 diabetes (%)					<0.001					0.025					0.581
No	92.4	87.6	86.9	84.8		88.5	89.0	85.1	89.5		87.7	87.2	88.0	89.4	
Yes	7.6	12.4	13.2	15.2		11.5	11.0	14.9	10.6		12.3	12.8	12.0	10.6	
Metabolic syndrome ^d^ (%)					<0.001					0.052					<0.001
<3 factors satisfied	69.3	78.8	86.0	86.2		77.8	81.9	81.5	77.9		82.7	82.3	81.2	72.0	
≥3 factors satisfied	30.7	21.2	14.0	13.8		22.2	18.1	18.5	22.1		17.3	17.7	18.8	28.0	
BMI (kg/m^2^, %)					0.028					0.681					0.004
Normal (BMI < 23)	32.4	32.5	34.6	33.3		32.5	34.2	33.5	32.6		34.4	35.4	32.2	30.2	
Overweight (23 ≤ BMI < 25)	24.5	24.7	29.5	28.1		28.6	27.2	25.6	25.5		28.5	27.1	27.0	23.4	
Obese (BMI ≥ 25)	43.1	42.8	35.9	38.6		39.0	38.6	40.9	41.9		37.1	37.5	40.7	46.4	
Energy intake (kcal/day)	1846.7	1626.3	1848.2	2267.5	<0.001	1764.1	1615.9	1769.0	2361.9	<0.001	1906.2	1647.2	1840.4	2215.1	<0.001
Carbohydrate intake (g/day)	355.7	297.9	320.3	378.4	<0.001	322.8	292.0	311.1	417.1	<0.001	331.9	298.4	331.1	398.4	<0.001
Protein intake (g/day)	55.7	52.7	64.7	83.5	<0.001	55.2	52.9	60.9	83.1	<0.001	65.2	53.7	61.6	76.0	<0.001
Fat intake (g/day)	20.8	22.6	32.3	45.2	<0.001	24.9	23.7	29.3	40.2	<0.001	33.8	24.3	27.8	33.9	<0.001

^a^*p*-values from the chi-square test for categorical variables and one-way ANOVA for continuous variables; ^b^ High income ≥840 dollar/month and low income <840 dollar/month (840 dollar is equivalent to 1 million Korean won); ^c^ Vigorous exercise <30 minutes/day and ≥30 minutes/day; ^d^ Metabolic syndrome is diagnosed if more than 3 factors are satisfied among the following components: (1) waist circumference ≥85 cm for women; (2) SBP ≥130 mmHg or DBP ≥85 mmHg; (3) TG levels ≥150 mg/dL; (4) HDL cholesterol levels <50 mg/dL for women; and (5) fasting plasma glucose levels ≥100 mg/dL.

**Table 3:** Hazard ratios (HRs) and 95% confidence intervals (CIs) for type 2 diabetes from multivariable Cox regression in female subjects.

Variables	Bread and Snacks		Meat and Fish		Korean Traditional	
	HR	95% CI	HR	95% CI	HR	95% CI
Age						
40–49 years		Ref ^a^		Ref		Ref
50–59 years	1.43	(1.10–1.86)	1.42	(1.09–1.85)	1.43	(1.10–1.87)
≥60 years	1.34	(0.96–1.88)	1.33	(0.95–1.85)	1.35	(0.96–1.88)
Marital status						
Lives with spouse		Ref		Ref		Ref
Single	0.94	(0.70–1.25)	0.95	(0.71–1.26)	0.94	(0.71–1.26)
Household income ^b^						
High income		Ref		Ref		Ref
Low income	0.81	(0.62–1.06)	0.80	(0.61–1.04)	0.79	(0.61–1.03)
Education						
Below high school graduate		Ref		Ref		Ref
Above high school graduate	1.06	(0.82–1.37)	1.09	(0.85–1.41)	1.08	(0.84–1.39)
Physical activity ^c^						
<30 min/day		Ref		Ref		Ref
≥30 min/day	0.90	(0.73–1.12)	0.91	(0.74–1.13)	0.91	(0.73–1.12)
Drinking						
Nondrinker		Ref		Ref		Ref
Drinker	1.12	(0.89–1.42)	1.14	(0.90–1.44)	1.13	(0.89–1.43)
Family history of type 2 diabetes						
No		Ref		Ref		Ref
Yes	1.61	(1.26–2.05)	1.63	(1.27–2.08)	1.62	(1.27–2.07)
Metabolic syndrome ^d^						
<3 factors satisfied		Ref		Ref		Ref
≥3 factors satisfied	2.60	(2.04–3.31)	2.55	(2.00–3.24)	2.58	(2.02–3.28)
BMI (kg/m^2^)						
Normal (BMI < 23)		Ref		Ref		Ref
Overweight (23 ≤ BMI < 25)	1.18	(0.86–1.62)	1.20	(0.87–1.65)	1.18	(0.86–1.62)
Obese (BMI ≥ 25)	1.55	(1.16–2.06)	1.56	(1.17–2.09)	1.54	(1.15–2.05)
Energy intake (100 kcal/day)	0.99	(0.97–1.02)	0.99	(0.97–1.01)	1.00	(0.98–1.02)
Quartiles of dietary pattern						
Quartile 1		Ref		Ref		Ref
Quartile 2	0.97	(0.72–1.32)	1.34	(0.99–1.82)	0.81	(0.59–1.12)
Quartile 3	1.08	(0.79–1.47)	1.31	(0.94–1.84)	1.00	(0.73–1.33)
Quartile 4	1.18	(0.84–1.66)	1.72	(1.28–2.32)	0.83	(0.60–1.15)

^a^ Ref stands for reference category; ^b^ High income ≥840 dollar/month and low income <840 dollar/month (840 dollar is equivalent to 1 million Korean won); ^c^ Vigorous exercise <30 minutes/day and ≥30 minutes/day; ^d^ Metabolic syndrome is diagnosed if more than 3 factors are satisfied among the following components: (1) waist circumference ≥85 cm for women; (2) SBP ≥130 mmHg or DBP ≥85 mmHg; (3) TG levels ≥150 mg/dL; (4) HDL cholesterol levels <50 mg/dL for women; and (5) fasting plasma glucose levels ≥100 mg/dL.

**Table 4:** Proportional hazards assumption test ^a^ in multivariable Cox regression.

Variables	Bread and Snacks	Meat and Fish	Korean Traditional
	*p*-Value	*p*-Value	*p*-Value
Age			
40–49 years	Ref ^b^	Ref	Ref
50–59 years	0.780	0.807	0.988
≥60 years	0.745	0.696	0.516
Marital status			
Lives with spouse	Ref	Ref	Ref
Single	0.132	0.126	0.183
Household income ^c^			
High income	Ref	Ref	Ref
Low income	0.107	0.150	0.214
Education			
Below high school graduate	Ref	Ref	Ref
Above high school graduate	0.657	0.414	0.261
Physical activity ^d^			
<30 min/day	Ref	Ref	Ref
≥30 min/day	0.882	0.633	0.777
Drinking			
Nondrinker	Ref	Ref	Ref
Drinker	0.672	0.639	0.702
Family history of type 2 diabetes			
No	Ref	Ref	Ref
Yes	0.302	0.320	0.350
Metabolic syndrome ^e^			
<3 factors satisfied	Ref	Ref	Ref
≥3 factors satisfied	0.037	0.043	0.049
BMI (kg/m^2^)			
Normal (BMI < 23)	Ref	Ref	Ref
Overweight (23 ≤ BMI < 25)	0.379	0.294	0.318
Obese (BMI ≥ 25)	0.203	0.199	0.286
Energy intake (100 kcal/day)	0.103	0.817	0.088
Quartiles of dietary pattern			
Quartile 1	Ref	Ref	Ref
Quartile 2	0.311	0.232	0.529
Quartile 3	0.859	0.034	0.002
Quartile 4	0.050	0.010	0.005
Global test	0.158	0.077	0.017

^a^ All values were estimated from scaled Schoenfeld residuals test; ^b^ Ref stands for reference category; ^c^ High income ≥840 dollar/month and low income <840 dollar/month (840 dollar is equivalent to 1 million Korean won); ^d^ Vigorous exercise <30 minutes/day and ≥30 minutes/day; ^e^ Metabolic syndrome is diagnosed if more than 3 factors are satisfied among the following components: (1) waist circumference ≥85 cm for women; (2) SBP ≥130 mmHg or DBP ≥85 mmHg; (3) TG levels ≥150 mg/dL; (4) HDL cholesterol levels <50 mg/dL for women; and (5) fasting plasma glucose levels ≥100 mg/dL.

**Table 5:** Hazard ratios (HRs) and 95% confidence intervals (CIs) for type 2 diabetes from multivariable stratified Cox regression with interaction terms of metabolic syndrome and meat and fish dietary patterns in female subjects.

Variables	Meat and Fish	
	HR	95% CI
Age		
40–49 years		Ref ^a^
50–59 years	1.12	(0.57–2.21)
≥60 years	0.95	(0.39–2.36)
Marital status		
Lives with spouse		Ref
Single	0.86	(0.39–1.89)
Household income ^b^		
High income		Ref
Low income	1.11	(0.55–2.25)
Education		
Below high school graduate		Ref
Above high school graduate	1.07	(0.58–1.98)
Physical activity ^c^		
<30 min/day		Ref
≥30 min/day	1.47	(0.85–2.54)
Drinking		
Nondrinker		Ref
Drinker	0.79	(0.42–1.48)
Family history of type 2 diabetes		
No		Ref
Yes	1.27	(0.65–2.48)
BMI (kg/m^2^)		
Normal (BMI < 23)		Ref
Overweight (23 ≤ BMI < 25)	0.86	(0.41–1.81)
Obese (BMI ≥ 25)	1.27	(0.66–2.45)
Energy intake (100 kcal/day)	0.96	(0.92–1.00)
Age ≥ 60 years		
Interaction with MS	0.60	(0.30–1.19)
Interaction with Q2	1.92	(0.73–5.03)
Interaction with Q3	2.60	(0.87–7.80)
Interaction with Q4	2.86	(1.12–7.33)
Obese (BMI ≥ 25)		
Interaction with MS	1.82	(1.01–3.26)
Interaction with Q2	1.36	(0.62–2.98)
Interaction with Q3	0.77	(0.34–1.75)
Interaction with Q4	0.92	(0.45–1.90)

Abbreviation: MS, metabolic syndrome; ^a^ Ref stands for reference category; ^b^ High income ≥840 dollar/month and low income <840 dollar/month (840 dollars is equivalent to 1 million Korean won); ^c^ Vigorous exercise <30 minutes/day and ≥30 minutes/day.

## References

[1] World Health Organization (2016). Global Report on Diabetes.

[2] Zhang P., Zhang X., Brown J., Vistisen D., Sicree R., Shaw J., Nichols G. (2010). Global healthcare expenditure on diabetes for 2010 and 2030. Diabetes Res. Clin. Pract..

[3] Alhazmi A., Stojanovski E., McEvoy M., Garg M.L. (2014). The association between dietary patterns and type 2 diabetes: A systematic review and meta-analysis of cohort studies. J. Hum. Nutr. Diet..

[4] Cox D.R. (1972). Regression models and life-tables. J. R Stat. Soc. Series B Stat. Methodol..

[5] Uno H., Claggett B., Tian L., Inoue E., Gallo P., Miyata T., Schrag D., Takeuchi M., Uyama Y., Zhao L. (2014). Moving beyond the hazard ratio in quantifying the between-group difference in survival analysis. J. Clin. Oncol..

[6] Jannasch F., Kröger J., Schulze M.B. (2017). Dietary patterns and type 2 diabetes: A systematic literature review and meta-analysis of prospective studies. J. Nutr..

[7] de Koning L., Chiuve S.E., Fung T.T., Willett W.C., Rimm E.B., Hu F.B. (2011). Diet-quality scores and the risk of type 2 diabetes in men. Diabetes Care.

[8] Otto M.C., Padhye N.S., Bertoni A.G., Jacobs D.R., Mozaffarian D. (2015). Everything in moderation—Dietary diversity and quality, central obesity and risk of diabetes. PLoS ONE.

[9] Pan W.H., Yeh W.T., Weng L.C. (2008). Epidemiology of metabolic syndrome in Asia. Asia Pac. J. Clin. Nutr..

[10] Qiao Y., Tinker L., Olendzki B.C., Hébert J.R., Balasubramanian R., Rosal M.C., Hingle M., Song Y., Schneider K.L., Liu S. (2014). Racial/ethnic disparities in association between dietary quality and incident diabetes in postmenopausal women in the United States: The Women’s Health Initiative 1993–2005. Ethn. Health.

[11] Zamora D., Gordon-Larsen P., He K., Jacobs D.R., Shikany J.M., Popkin B.M. (2011). Are the 2005 Dietary guidelines for Americans associated with reduced risk of type 2 diabetes and cardiometabolic risk factors? Twenty-year findings from the CARDIA study. Diabetes Care.

[12] Korean Diabetes Association (2018). Diabetes Fact Sheet in Korea. http://www.diabetes.or.kr/pro/news/admin.php?category=A&code=admin&number=1615&mode=view.

[13] Lim J.H., Lee Y.S., Chang H.C., Moon M.K., Song Y. (2011). Association between dietary patterns and blood lipid profiles in Korean adults with type 2 diabetes. J. Korean Med. Sci..

[14] Vatcheva K.P., Lee M., McCormick J.B., Rahbar M.H. (2015). The effect of ignoring statistical interactions in regression analyses conducted in epidemiologic studies: An example with survival analysis using Cox proportional hazards regression model. Epidemiology (Sunnyvale Calif.).

[15] Kim Y., Han B.G., KoGES Group (2017). Cohort profile: The Korean Genome and Epidemiology Study (KoGES) consortium. Int. J. Epidemiol..

[16] Kim H.L., Kim M.A., Park K.T., Choi D.J., Han S., Jeon E.S., Cho M.C., Kim J.J., Yoo B.S., Shin M.S. (2019). Gender difference in the impact of coexisting diabetes mellitus on long-term clinical outcome in people with heart failure: A report from the Korean Heart Failure Registry. Diabetes Med..

[17] Ahn Y., Kwon E., Shim J.E., Park M.K., Joo Y., Kimm K., Park C., Kim D.H. (2007). Validation and reproducibility of food frequency questionnaire for Korean genome epidemiologic study. Eur. J. Clin. Nutr..

[18] Grundy S.M., Cleeman J.I., Daniels S.R., Donato K.A., Eckel R.H., Franklin B.A., Gordon D.J., Krauss R.M., Savage P.J., Smith S.C. (2005). Diagnosis and management of the metabolic syndrome: An American Heart Association/National Heart, Lung, and Blood Institute Scientific Statement. Circulation.

[19] Lee S.Y., Park H.S., Kim D.J., Han J.H., Kim S.M., Cho G.J., Kim D.Y., Kwon H.S., Kim S.R., Lee C.B. (2007). Appropriate waist circumference cutoff points for central obesity in Korean adults. Diabetes Res. Clin. Pract..

[20] Grambsch P.M., Therneau T.M. (1994). Proportional hazards tests and diagnostics based on weighted residuals. Biometrika.

[21] Stata manuals 13 (2013). Stcox PH-Assumption Tests—Tests of Proportional-Hazards Assumption. https://www.stata.com/manuals13/ststcoxph-assumptiontests.pdf.

[22] Kleinbaum D., Klein M. (2011). Survival Analysis: A Self-Learning Text (Statistics for Biology and Health).

[23] Woo H.D., Shin A., Kim J. (2014). Dietary patterns of Korean adults and the prevalence of metabolic syndrome: A cross-sectional study. PLoS ONE.

[24] Odegaard A.O., Koh W.P., Butler L.M., Duval S., Gross M.D., Yu M.C., Yuan J.M., Pereira M.A. (2011). Dietary patterns and incident type 2 diabetes in Chinese men and women: The Singapore Chinese health study. Diabetes Care.

[25] Nanri A., Shimazu T., Takachi R., Ishihara J., Mizoue T., Noda M., Inoue M., Tsugane S., Japan Public Health Center-based Prospective Study Group (2013). Dietary patterns and type 2 diabetes in Japanese men and women: The Japan Public Health Center-based Prospective Study. Eur. J. Clin. Nutr..

[26] InterAct Consortium (2014). Adherence to predefined dietary patterns and incident type 2 diabetes in European populations: EPIC-InterAct Study. Diabetologia.

[27] von Ruesten A., Illner A.K., Buijsse B., Heidemann C., Boeing H. (2010). Adherence to recommendations of the German food pyramid and risk of chronic diseases: Results from the EPIC-Potsdam study. Eur. J. Clin. Nutr..

[28] Lorenzo C., Okolois M., Williams K., Stern M.P., Haffner S.M., San Antonio Heart Study (2003). The metabolic syndrome as predictor of type 2 diabetes: The San Antonio heat study. Diabetes Care.

[29] Patino-Alonso M.C., Recio-Rodríguez J.I., Belio J.F., Colominas-Garrido R., Lema-Bartolomé J., Arranz A.G., Agudo-Conde C., Gomez-Marcos M.A., García-Ortiz L., EVIDENT Group (2014). Factors associated with adherence to the Mediterranean diet in the adult population. J. Acad. Nutr. Diet..

[30] Fransen H.P., Boer J.M.A., Beulens J.W.J., de Wit G.A., Bueno-de-Mesquita H.B., Hoekstra J., May A.M., Peeters P.H.M. (2017). Associations between life factors and an unhealthy diet. Eur. J. Public Health.

[31] Montonen J., Boeing H., Fritsche A., Schleicher E., Joost H.G., Schulze M.B., Steffen A., Pischon T. (2013). Consumption of red meat and whole-grain bread in relation to biomarkers of obesity, inflammation, glucose metabolism and oxidative stress. Eur. J. Nutr..

[32] Ley S.H., Sun Q., Willett W.C., Eliassen A.H., Wu K., Pan A., Grodstein F., Hu F.B. (2014). Associations between red meat intake and biomarkers of inflammation and glucose metabolism in women. Am. J. Clin. Nutr..

[33] Lastra G., Syed S., Kurukulasuriya L.R., Manrique C., Sowers J.R. (2014). Type 2 diabetes mellitus and hypertension: An update. Endocrinol. Metab. Clin. N. Am..

[34] Shim J.S., Oh K., Kim H.C. (2014). Dietary assessment methods in epidemiologic studies. Epidemiol. Health.

[35] Kim K.N., Oh S.Y., Hong Y.C. (2018). Associations of serum calcium levels and dietary calcium intake with incident type 2 diabetes over 10 years: The Korean Genome and Epidemiology Study (KoGES). Diabetol. Metab. Syndr..

[36] Kim J., Oh B., Lim J.E., Kim M.K. (2016). No Interaction with Alcohol Consumption, but Independent Effect of C12orf51 (HECTD4) on Type 2 Diabetes Mellitus in Korean Adults Aged 40–69 Years: The KoGES_Ansan and Ansung Study. PLoS ONE.

[37] Royston P., Parmer M.K. (2013). Restricted mean survival time: An alternative to the hazard ratio for the design and analysis of randomized trials with a time-to-event outcome. BMC Med Res. Methodol..

